# Novel Cardiac Computed Tomography Methods for the Assessment of Anthracycline Induced Cardiotoxicity

**DOI:** 10.3389/fcvm.2022.875150

**Published:** 2022-04-27

**Authors:** Attila Feher, Lauren A. Baldassarre, Albert J. Sinusas

**Affiliations:** ^1^Department of Internal Medicine, Section of Cardiovascular Medicine, Yale University School of Medicine, New Haven, CT, United States; ^2^Department of Radiology and Biomedical Imaging, Yale University School of Medicine, New Haven, CT, United States; ^3^Department of Biomedical Engineering, Yale University, New Haven, CT, United States

**Keywords:** computed tomography, anthracycline, doxorubicin, cardiotoxicity, cardiooncology, cardiovascular computed tomography, strain, fibrosis

## Abstract

Anthracyclines are among the most frequently utilized anti-cancer therapies; however, their use is frequently associated with off-target cardiotoxic effects. Cardiac computed tomography (CCT) is a validated and rapidly evolving technology for the evaluation of cardiac structures, coronary anatomy and plaque, cardiac function and preprocedural planning. However, with emerging new techniques, CCT is rapidly evolving to offer information beyond the evaluation of cardiac structure and epicardial coronary arteries to provide details on myocardial deformation, extracellular volume, and coronary vasoreactivity. The potential for molecular imaging in CCT is also growing. In the current manuscript we review these emerging computed tomography techniques and their potential role in the evaluation of anthracycline-induced cardiotoxicity.

## Introduction

Cancer is the second leading cause of death in the United States. Approximately 1.9 million new cancer cases and 600,000 cancer deaths are projected to occur in the United States in 2021 (1). As of 2019, there were an estimated 16.9 million cancer survivors living in the United States and this number is projected to increase to 22.2 million by 2030 (2). In view of the significant improvement in survival, cardiovascular health has become an emerging focus of interest in cancer survivals.

Anthracyclines are among the most frequently utilized anti-cancer agents and they rank high among the most effective antineoplastic therapies (3). The first anthracycline, daunorubicin was discovered in the early 1960s by Di Marco et al. who isolated daunorubicin from a strain of *Streptomyces peucetius* (4). Shortly, this was followed by the discovery of other anthracycline agents which all demonstrated anti-cancer properties, including the most frequently used anthracyclines in addition to daunorubicin: doxorubicin, idarubicin, epirubicin and mitoxantron. The exact mechanism of anthracycline effect on cancer cells remains unclear, however it is likely multifactorial. The following mechanisms have been suggested (1) intercalation into deoxyribonucleic acid (DNA), leading to inhibited protein synthesis; (2) free radical generation leading to inflammation, lipid peroxidation, and DNA damage; (3) DNA binding, alkylation or cross-linking and/or interference with DNA unwinding or DNA strand separation and helicase activity; (4) direct membrane and other cellular cytotoxic effects; and (5) topoisomerase II inhibition leading to DNA damage and apoptosis (3). Despite the fact that it has been almost 60 years since the discovery of anthracyclines, and despite the recent unprecedented breakthrough in cancer treatment achieved by targeted oncologic therapies, about 30% of breast cancer patients, 50 to 70% of elderly lymphoma patients, and up to 50 to 60% of childhood cancer patients are treated with an anthracycline based regimen (5).

**FIGURE 1:**
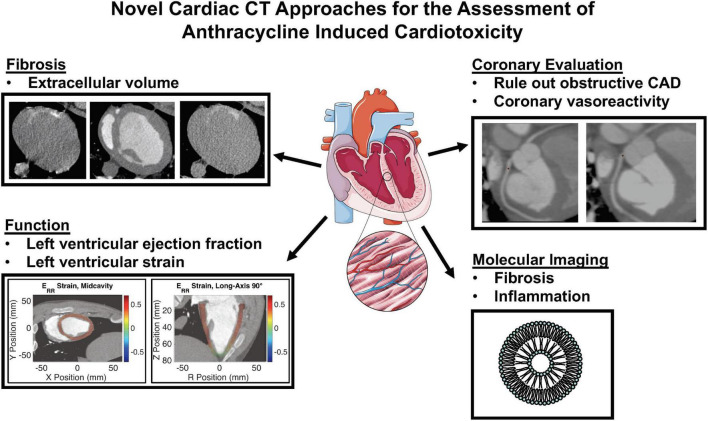
Novel cardiac computed tomography (CT) approaches for the assessment of anthracycline induced cardiotoxicity. CAD, coronary artery disease.

Cardiac computed tomography (CCT) is a validated, rapidly evolving technology for the evaluation of cardiac structures with or without contrast administration, including evaluation of coronary anatomy and coronary atherosclerotic disease with high spatial resolution, evaluation of cardiac function and preprocedural planning for electrophysiologic and percutaneous valvular interventions. This review aims to summarize the current and future CCT applications that could be used for pre-treatment risk evaluation, cardiotoxicity surveillance and early identification during anthracycline therapy (Figure 1). Although CCT has value for the evaluation of cancer therapy associated valvular heart disease, pericardial disease and primary/secondary malignancies involving the heart, discussion of these applications is beyond the scope of this review.

## Mechanism of Anthracycline Induced Cardiotoxicity

Anthracyclines have become one of the most effective antineoplastic agents, however, it was recognized relatively early that their use is associated with increasing incidence of heart failure (6, 7). Importantly, one of these early studies demonstrated the dose dependent association between the most commonly used anthracycline, doxorubicin, and cardiotoxicity, namely that the incidence of clinical heart failure was exponentially higher in patients who were exposed to doxorubicin over a cumulative dose of 550 mg/m^2^ (7). This has been confirmed by a later retrospective pooled cohort study showing that the incidence of heart failure varies with exposed cumulative dose of anthracyclines: 4.7% at 400 mg/m^2^, 26% at 550 mg/m^2^ and 48% at 700 mg/m^2^ (8). Importantly, the incidence of anthracycline induced cardiotoxicity (AIC) is influenced by many other factors including genetic variability, age (with children and the elderly at higher risk), previous treatment with cardiotoxic drugs or radiation therapy, and history of cardiovascular disease (9).

The mechanisms responsible for AIC are complex and incompletely understood. Initial observations linked doxorubicin induced cardiotoxicity to the formation of reactive oxygen species (ROS) leading to myocyte damage by oxidative injury (10–13). Myocardial injury by complex formation of anthracyclines with topoisomerase IIb causing double-stranded DNA breaks has been more recently proposed as an alternative main mechanism for AIC (5).

Cardiomyocytes comprise 80% of the cardiac mass, however they only account for less than 20% of the cardiac cells. Other cells, such as endothelial cells, smooth muscle cells, fibroblasts and adipocytes provide structural and functional support and form a unique environment for the cardiomyocytes. Emerging evidence suggests that the deleterious effect of anthracyclines is not limited to cardiomyocytes. As such, anthracycline administration has been linked to microvascular injury by direct endothelial DNA damage (14), by promoting apoptosis through oxidative stress, (15, 16) or by interfering with endothelial nitric oxide bioavailability and nitric oxide signaling (17). Related to this, in rodent models, AIC could be rescued by targeting endothelial inflammation or angiogenesis (18, 19). A recent review concluded that the microvascular endothelium serves an important novel target for the early detection, prevention, and treatment of AIC (20).

The extracellular matrix plays a unique role in cardiac homeostasis, not only by providing structural support, but also by facilitating force transmission and cell to cell communication. Although there are multiple pathways that contribute to anthracycline induced cardiac injury, ultimately all these processes lead to a common downstream pathway that results in alteration of proteolytic pathways leading to disruption in the very fine balance of myocardial matrix metalloproteinases and their inhibitors resulting in deposition of components of the extracellular matrix and tissue fibrosis (21–23).

## Evaluation of Left Ventricular Systolic Function and Strain: Role of CT in Assessment of Anthracycline Induced Cardiotoxicity

Multi-phase cardiac computed tomography (CT) with retrospective ECG gating allows for 3-dimensional analysis of the cardiac function with high spatial resolution. Advances in CT technology have resulted in lower radiation dose and higher temporal resolution for cardiac imaging techniques (24). Electrocardiogram pulsing reduces radiation by modulating the tube current during the scan, applying higher amount of tube current during diastole with lower tube current during the rest of the cardiac cycle (25).

Computed tomography based evaluation of left ventricular volumes has been reported as early as 2004, (26); however, the routine use of CT for left ventricular systolic function evaluation has been limited due to the elevated radiation dose associated with retrospectively gated studies.

Left ventricular global longitudinal strain (GLS) as assessed by 2-dimensional transthoracic echocardiography is a well described early predictor of AIC (27, 28). LV GLS is a more sensitive, reproducible measure of LV dysfunction when compared to LVEF. In a recent meta-analysis, measurement of echocardiographic GLS after initiation of anthracycline based chemotherapy with or without trastuzumab had good prognostic performance for predicting subsequent cancer therapy-related cardiac dysfunction defined as clinically significant change in left ventricular ejection fraction (LVEF) with or without new-onset heart failure symptoms (27). The recently published SUCCOR (Strain sUrveillance of Chemotherapy for improving Cardiovascular Outcomes) international multicenter prospective randomized controlled trial evaluated whether echocardiographic GLS guided cardioprotective therapy would prevent reduction in LVEF and development of chemotherapy induced cardiotoxicity in comparison to standard, LVEF guided care in a high risk population receiving cardiotoxic chemotherapy (29). Despite the fact that the primary outcome, defined as change in LVEF was not significantly different between the 2 arms at 1 year follow-up, in the GLS-guided group fewer patients met the prespecified criteria for chemotherapy induced cardiotoxicity compared to the LVEF-guided group. The LVEF at 1 year follow-up approached achieved a significant difference for the LVEF-guided versus the GLS-guided groups (55 ± 7% versus 57 ± 6%, *p* = 0.05) (29). In this trial, restricting the analysis to cancer patients who received cardioprotective therapy, the LVEF-guided arm had significantly more reduction in LVEF on follow-up echocardiogram when compared to GLS-guided arm (9 ± 11% vs. 3 ± 7%, *p* = 0.03).

**FIGURE 2:**
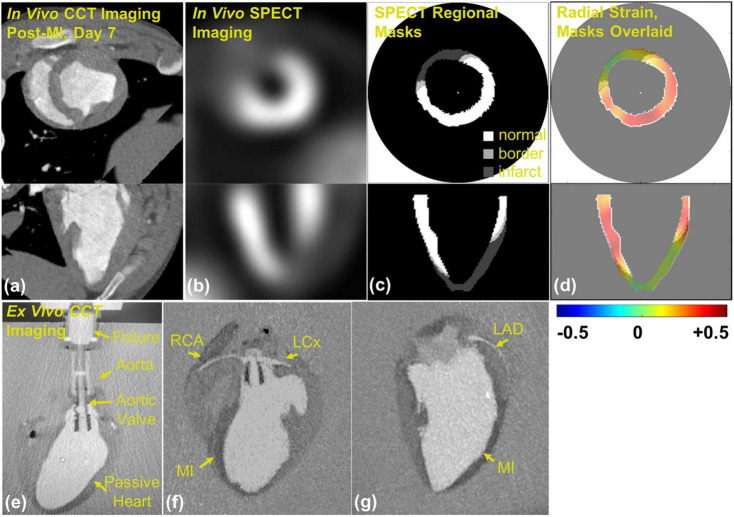
Contrast CineCT and 99mTc-Tetrofosmin SPECT imaging and masking in porcine heart: (a) *in vivo* contrast CineCT short- and long-axis imaging of porcine heart on day 7 after myocardial infarction (MI), (b) *in vivo* SPECT short- and long-axis imaging of the porcine heart on day 7 after MI, (c) SPECT-derived regional masks for strain fields of normal (white, > 60% max intensity), border (light gray, > 50% and < 60% max intensity), and infarct (dark gray, < 50% max intensity) regions, and (d) SPECT strain masks overlaid on radial strain field. *Ex vivo* contrast CineCT imaging of the arrested porcine heart 7 after hydrogel delivery: (e) custom aortic valve insert and suspension fixture, (f) MI region and perfusion of right coronary artery (RCA) and left circumflex coronary artery (LCx), and (g) MI region and left anterior descending coronary artery (LAD). Note: this image is reproduced with permission from Midgett et al. (33).

The possibility of quantitative assessment of regional myocardial deformation from cine CT was demonstrated by Shi et al. using shape-based tracking of the ventricular surface (30) and has been subsequently reported and validated by several other groups (31, 32). Tissue tracking is used to calculate strain between sequential image frames, from systole to diastole, by using techniques adapted from echocardiography and cardiac MRI (30). The feasibility of performing CT strain has been demonstrated in large animal studies (Figure 2) (33) and in humans (34–37). A recent study assessing 44 heart failure patients undergoing both ECG gated cardiac CT and cardiac MRI within 24 h, showed good reproducibility of CT strain measurements and detected a good correlation between CT global longitudinal strain (GLS) and MRI GLS (38). In patients undergoing transcatheter aortic valve replacement CT strain was predictive of adverse outcomes, (36) and improvement in CT derived global longitudinal and principal strain has been demonstrated after transcatheter aortic valve replacement (34). The optimal evaluation of regional strain from CT images should employ 3-dimensional tracking of regional myocardial displacements (37), and probably will employ deep learning (39). Automated segmentation of left ventricular cavity in temporal cardiac image sequences (consisting of multiple time-points) is a fundamental requirement for quantitative analysis of cardiac structural and functional changes. A recently published approach employed a spatial-sequential network with bi-directional learning of 4D CT images which out-performed existing approaches for automated LV segmentation (40). Currently, there are commercially available programs for calculation of regional myocardial strain from contrast cine CT but these are based on 2D analysis of stacks of images (41). Although CT imaging holds promise for true high-resolution 3D analysis of regional myocardial strain, to date CT derived strain has not yet been assessed as a tool for the early prediction of AIC.

## CT Evaluation of Epicardial and Microvascular Coronary Injury

Cardiac CT plays a central role in the evaluation of coronary artery disease (CAD) in patients presenting with chest pain syndromes. The recent chest pain guideline specifies the use of coronary CT angiography (CTA) as a Class I recommendation for the exclusion of atherosclerotic plaque and obstructive CAD in intermediate-risk patients with acute chest pain and no known CAD (42). Coronary CT angiography also meets Class I recommendation for diagnosis of CAD, risk stratification and guiding treatment decisions for patients with stable chest pain who have an intermediate to high risk of obstructive CAD. Pre-existing CAD is known to increase the risk of development of cardiovascular complications in patients receiving anthracycline-based chemotherapy or receiving chest irradiation as a part of anti-cancer therapy (43), however current guidelines do not recommend routine assessment for evidence of CAD prior to initiation of anthracycline based chemotherapy. On the other hand, heart failure guidelines recommend the exclusion of CAD in patients with a new diagnosis of heart failure with reduced ejection fraction (44). Coronary CTA has been demonstrated to be a highly sensitive tool for the detection of obstructive CAD in patients with dilated cardiomyopathy (45, 46). Therefore, coronary CTA might be utilized to exclude epicardial coronary stenosis in patients with prior anthracycline exposure and newly developed reduced left ventricular ejection fraction. In a single center retrospective study, coronary CTA findings altered the therapeutic plan in 52% of 80 cancer patients undergoing coronary CTA by aiding in the decision of withholding, altering or continuing oncologic therapy (47). Coronary CTA can be used to rule out obstructive CAD in cancer patients with increased troponin levels after undergoing anthracycline treatment, which can be observed in up to 30% of patients receiving high dose chemotherapy (48). In addition, coronary CTA can be particularly helpful with the initial evaluation of patients presenting with chest pain and concurrent severe thrombocytopenia (relatively common side effect of anthracyclines), when invasive evaluation is often not feasible with relative contraindication for the use of heparin containing products. In addition, CT angiography can be further utilized for the evaluation of venous and arterial thrombosis, which are emerging new contributors to the development of AIC (49).

Coronary artery calcium (CAC) scoring by non-contrast CT provides a marker of CAD burden within the epicardial coronary arteries (50). The presence of CAC is a strong predictor of future cardiovascular risk with higher prognostic value than traditional risk assessment tools such as the Framingham risk score and the Atherosclerotic Cardiovascular Disease (ASCVD) risk score (50, 51). Moreover, CAC quantification has been demonstrated to be feasible on non-gated, non-cardiac chest CT scans with excellent correlation with standard CAC score derived from gated cardiac acquisitions (52). El-Sabbagh et al. demonstrated an average increase of 35% in non-gated CAC score in 112 lymphoma patients undergoing chemotherapy when comparing CAC score on pre and post chemotherapy non-gated CT examinations (53). Automated CAC scoring on non-gated non-contrast CT scans could be used as a fast and low-cost tool to identify cancer patients at higher cardiovascular risk, allowing implementation of cardiovascular risk reduction strategies prior to initiation of anthracycline based therapy. Importantly, the recently published joint guidelines the Society of Cardiovascular Computed Tomography and the Society of Thoracic Radiology recommends visual estimation for presence of CAC and encourages computation of a non-gated CAC score for all non-contrast chest CT examinations (54).

**FIGURE 3:**
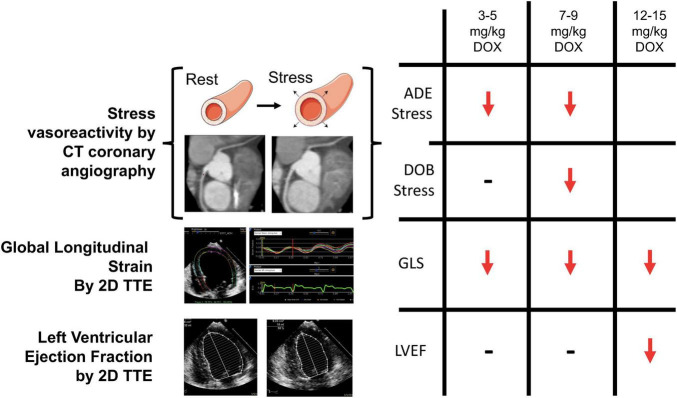
Computed tomography (CT) based assessment of coronary vasoreactivity in a canine model of doxorubicin (DOX) induced cardiotoxicity. Left ventricular ejection fraction (LVEF) was not reduced until a cumulative DOX dose of 12 to 15 mg/kg was administered, whereas impairment in ADE-induced vasodilator responses occurred early in the progression of DOX-induced cardiotoxicity similar to impairment in global longitudinal strain (GLS). 2D = 2-dimensional; TTE = transthoracic echocardiography. Note: this image is reproduced with permission from Feher et al. (28).

Recently, our group has developed a novel CT based strategy for the evaluation of coronary microvascular dysfunction associated with AIC (28). In this study, we used a canine model of doxorubicin-induced cardiotoxicity, where canines received weekly intravenous doxorubicin (1 mg/kg) for 12–15 weeks resulting in significant reduction in the left ventricular ejection fraction and histological evidence of cardiotoxicity by the end of therapy. Epicardial coronary artery diameters were measured at pre-specified distances from vessel origins from coronary CTA performed during rest, and in the presence of adenosine and dobutamine stress. Adenosine vasodilator responses (increase in epicardial coronary diameter) were impaired after ∼4 mg/kg and ∼8 mg/kg cumulative doxorubicin dosing, whereas dobutamine induced dilation response was preserved at ∼4 mg/kg, but tended to decrease at ∼8 mg/kg of doxorubicin (Figure 3). A significant LVEF reduction was observed only at 12–15 mg/kg doxorubicin dosing. These findings suggest an early impairment in microvascular responses in AIC. Adenosine-induced epicardial coronary responses depend on a direct vasodilatory effect on vascular smooth muscle and on subsequent flow-mediated shear stress endothelial dependent vasodilation due to local endothelial nitric oxide secretion. In contrast, dobutamine-induced vasodilation is mostly a result of direct stimulation of myocardial and vascular β-adrenergic receptors. Our results may indicate the susceptibility of the endothelium to anthracyclines leading to an early impairment in endothelium-dependent coronary vasodilation.

## CT Evaluation of Extracellular Volume in Anthracycline-Induced Cardiotoxicity

Assessment and quantification of myocardial late gadolinium enhancement (LGE) using magnetic resonance imaging (MRI) has been well validated for quantitative evaluation of focal myocardial fibrosis in the setting of myocardial infarction and non-ischemic cardiomyopathy (55–58). With cardiac MRI the extracellular volume (ECV) of the myocardium can be estimated by measuring both blood and myocardial longitudinal (T1) relaxation times pre and post gadolinium administration, using the following formula:

$$E\,C\,V\,M\,R\,I=\left(1-h\,e\,m\,a\,t\,o\,c\,r\,i\,t\right)\,\frac{\frac{1}{\text{T}\,1\,\mathrm{myo}\,\mathrm{post}}\,-\,\frac{1}{\text{T}\,1\,\mathrm{myo}\,\mathrm{pre}}\,}{\frac{1}{\text{T}\,1\,\mathrm{blood}\,\mathrm{post}}\,-\,\frac{1}{\text{T}\,1\,\mathrm{blood}\,\mathrm{pre}}}$$

where, T1 myo and T1 blood represent the myocardial and blood T1 values before (pre) and after (post) gadolinium contrast administration. Several studies reported on excellent correlation between myocardial ECV assessed by MRI and quantitative histopathology (59–61). Importantly, multiple MRI studies have demonstrated that the ECV was elevated in patients treated with anthracyclines compared to matched control populations (62–64). Based on the observed association between increased ECV and decrease in intracellular water lifetime (a marker of cardiomyocyte size) and LV mass in response to anthracycline based chemotherapy, one of these studies suggested that in anthracycline-induced cardiac injury the increase in ECV may be due to a decrease in LV mass from cardiomyocyte loss rather than interstitial fibrosis and edema (64). This hypothesis will require further investigation. MRI has been invaluable for the evaluation of changes in ECV in AIC; however, there are minor drawbacks with the use of MRI for the evaluation of ECV, including: (1) prolonged examination protocols especially with the need of pre and post contrast T1 mapping, (2) associated increased medical cost, and (3) inability to image patients with claustrophobia.

In the last decade, CT has emerged as an additional tool for the assessment of cardiac fibrosis. The evaluation of ECV by CT relies upon the same principle as assessment with MRI. The CT imaging protocol requires acquisition of a low dose ECG-gated cardiac CT prior to contrast administration and repeat imaging following administration of contrast at a delayed timepoint, usually 10 min after iodinated contrast administration (65). The CT derived estimate of ECV is obtained using the following formula:

$$E\,C\,V\,C\,T=\left(1-h\,e\,m\,a\,t\,o\,c\,r\,i\,t\right)\,\frac{\Delta \,\mathrm{HU}\,\mathrm{myo}}{\Delta \,\mathrm{HU}\,\mathrm{blood}}$$

where, Δ HU myo and Δ HU blood are the change in Hounsfield unit attenuation pre- and post-contrast administration (e.g., HU post-contrast – HU pre-contrast) in the myocardium and the blood, respectively. ECV estimated by CT has been shown to correlate well with both MRI derived ECV (65–67) and pathological indices of fibrosis (66). Moreover CT derived ECV has been shown to be prognostic of adverse cardiovascular events in aortic stenosis in patients undergoing periprocedural CT evaluation (68).

**FIGURE 4:**
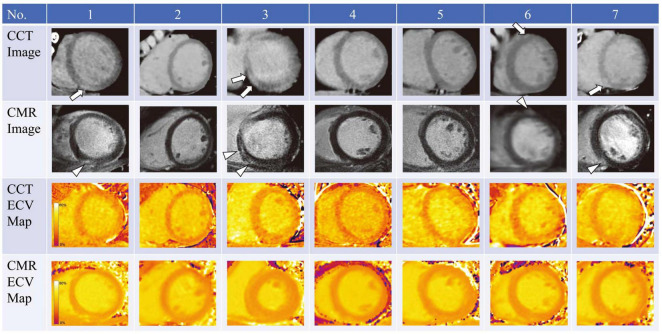
Cardiac computed tomography (CCT) and cardiac magnetic resonance (CMR) imaging in 7 patients who underwent anthracycline treatment. Arrows and arrowheads indicate late iodine and gadolinium enhancements, respectively. ECV, extracellular volume. Note: this image is reproduced with permission from Sueta et al. (74).

A handful of studies have investigated the use of ECV CT index in the assessment of AIC (69–74). Zhou et al. compared ECV assessed by CT and MRI in a chronic canine model of AIC (70). In this study canines were administered intravenous doxorubicin every 3 weeks achieving a cumulative dose of 240 mg/m^2^ and had follow-up CT and MRI examination at 16 and 24 weeks. CT detected an increase in ECV over time following doxorubicin administration (baseline: 25.2%, 16 weeks: 34.4%, 24 weeks: 37.7%). In this study ECV CT correlated well with both MRI ECV and with indices of fibrosis on histological analysis. In addition to animal models of AIC, a few studies have also looked at CT derived ECV in cancer patients receiving anthracycline-based chemotherapy regimens (71–74). In a small study, Sueta et al. found the ECV CT (33.0 ± 2.5%) elevated in 7 cancer patients with documented AIC which was confirmed by CMR assessment of ECV (Figure 4) (74). Another study evaluated 44 patients who previously received anthracycline based chemotherapy regimens, and found significantly higher ECV CT index in the patients with documented AIC (*n* = 7, ECV: 30.3 ± 4.8%) versus patients who received anthracycline without cardiac dysfunction (*n* = 37, ECV: 27.5 ± 3.1%) or control patients who did not receive cardiotoxic chemotherapy (*n* = 20, ECV: 26.2 ± 2.5%) (73). Monti et al. performed a retrospective study evaluating changes in ECV from serial thoracic non-gated contrast CT scans in 32 female patients with breast cancer who had examination before chemotherapy and repeat examination after completion of anthracycline based chemotherapy regimen (ECV was derived from non-gated pre-contrast and delayed phase scans obtained at 7 min post contrast injection) (71). The authors found increased ECV values post therapy (30.0 ± 5.1%) when compared to pre-treatment ECV values (26.4 ± 3.8%).

**FIGURE 5:**
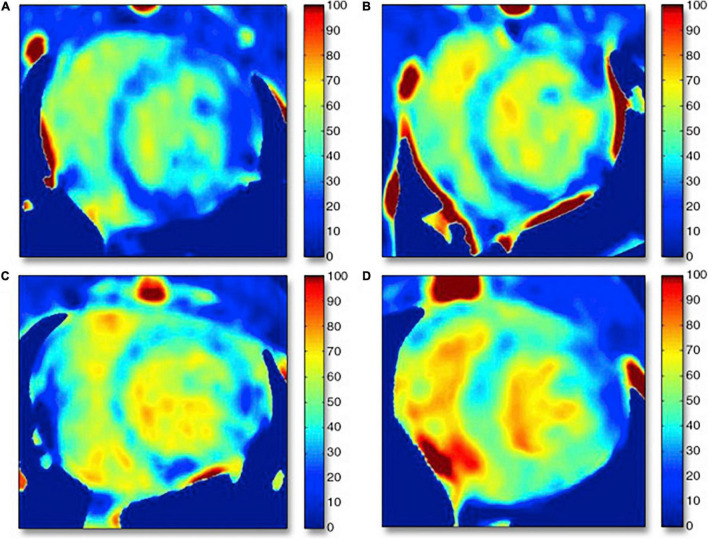
Examples of computed tomography extracellular volume (ECV) maps in control rabbits and rabbits undergoing anthracycline-based chemotherapy generated by dual energy CT acquisition. **(A)** Control subject (Hct = 44.4%), ECV = 27.0%; dark blue normal myocardium shown on the ECV map. **(B)** Six-week treatment model (Hct = 37.4%), ECV = 36.4%; dark blue septal wall changed to bright blue. **(C)** Twelve-week model (Hct = 30.0%), ECV = 44.0%; dark blue area of inferoseptal/inferolateral wall changed to bright blue myocardium. **(D)** Sixteen-week model (Hct = 25.0%), ECV = 46.0%; bright blue myocardium suggests myocardial fibrosis. Hct = hematocrit. Note: this image is reproduced with permission from Hong et al. (69).

The traditional contrast equilibrium method for ECV calculation has some potential technical challenges: (1) this approach requires both pre and post contrast scanning which despite the low dose nature of these scans, increases radiation dose, (2) difficulty in left ventricular segmentation of pre-contrast images, and (3) difficulty in the registration of the pre- and post-contrast images. However, dual source dual energy CT imaging offers a potential solution to these problems by performing a single scan with two orthogonally mounted detectors and tube arrays. This method can not only help with reducing beam hardening and metallic artifacts, but can potentially be used for quantifying ECV with the potential of eliminating the requirement of pre-contrast images reducing problems with mis-registration as well as reducing radiation dose. Hong et al. evaluated ECV by dual energy CTA in a rabbit model of dilated cardiomyopathy, generated by administering 1.0 mg/kg of doxorubicin twice weekly for 16 weeks (69). The mean ECV values were significantly higher after doxorubicin administration (baseline: 28.5%, 6 weeks: 35.3%, 12 weeks: 41.9% and 16 weeks: 42.1%) (Figure 5). ECV obtained with dual energy CT correlated remarkably very well with ECV estimated by CMR and fibrosis extent on histology. Zhao et al. also compared the use of dual energy CT with single energy CT for the estimation of ECV in a canine model of doxorubicin induced cardiotoxicity (75). Dual energy CT ECV analysis was performed by using iodine maps generated from delayed CT images acquired at 100 and 140 kVp. As the authors hypothesized, the ECV CT index derived by dual energy CT correlated well with ECV estimated with single energy CT at 100 kVp and with histological analysis.

## CT Molecular Imaging and Theranostics

Advancements in basic science and innovations in technology have led to a deeper understanding of the molecular and cellular processes that contribute to the pathophysiology of AIC. Molecular imaging, defined as the visualization, characterization, and non-invasive measurement of biological processes at the molecular and cellular level, has the ability to translate advancements in basic science to humans to facilitate early diagnosis, improve prognostication and guide targeted therapy across the spectrum of cardiovascular disease. Several molecular probes have been evaluated to interrogate molecular mechanisms which have been implicated in the pathophysiology of AIC by using predominantly radiolabeled imaging probes. The higher sensitivity of these radiolabeled probes makes nuclear imaging techniques more suitable for cardiac molecular imaging, however the wide availability of the clinical CT scanners and the fast scanning times coupled with high spatial resolution make CT a promising alternative approach for certain molecular imaging targets with high receptor density.

The currently used iodine-based contrast agents provide excellent tissue contrast for anatomic evaluation; however, these agents show rapid blood clearance and non-specific tissue distribution, both of which limit their use for targeted imaging. To overcome these issues, novel nanoparticles (1–100 nm diameter) have recently been assembled that incorporate high payloads of iodinated or inorganic contrast agents that may also use specific peptides or antibodies for improved sensitivity to detect molecular/cellular targets, while also improving signal-to-noise ratio (76). Moreover, theranostic platforms have also been designed that combine diagnostic properties with the capability of targeted delivery of therapies. As an example Zhu et al. constructed a unique theranostic platform for targeted chemotherapy and *in vitro* cancer cell imaging based on dendrimer-entrapped gold nanoparticles (CT contrast agent) conjugated with doxorubicin (77). Similarly, Lin et al. developed a β-cyclodextrin based micelle system which was successfully loaded with gold nanoparticles and doxorubicin achieving high drug delivery and favorable imaging properties (78). In the future these and similar theranostic agents could be potentially used for pre-clinical investigations to further enhance our understanding of AIC. Nuclear molecular imaging techniques have already been successfully applied to track inflammation by detecting reactive oxygen species formation (12) and matrix metalloproteinase activity (23) in animal models of AIC. Imaging these processes by CT based molecular imaging probes, as well as CT based imaging of molecular processes involved in the pathophysiology of AIC, such as apoptosis, fibrosis and angiogenesis holds great promise for the future. Specifically, pre-clinical molecular imaging with CT has already been applied for the imaging of fibrosis by targeting collagen (79), or imaging inflammation by targeting E-selectin (80).

Nanoparticulate CT agents have been applied successfully in multiple preclinical models in a wide variety of cardiovascular diseases, however the full clinical potential of these probes will not be achieved until these barriers can be overcome, in particular the issue of sensitivity. Multiple liposomal formulations without imageable properties assembled for drug delivery have been successfully translated to clinical applications and used in early phase clinical trials for the delivery of anti-cancer, anti-fungal, anti-inflammatory drugs and for the delivery of therapeutic genes. The use of this nanoparticulate therapy provides local delivery of high therapeutic doses, potentially minimizing systemic toxicity, and this topic has been reviewed extensively (81). Several clinical trials have demonstrated favorable pharmacokinetic and pharmacodynamic profiles for these liposomal agents, along with excellent bioavailability and most importantly favorable safety profile in humans. An intravenously administered product named PEGylated liposomal iodixanol injection (NCTX), which has been previously tested in small and large animal models (82), has entered phase 1 tolerability and pharmacokinetic study in healthy volunteers (ClinicalTrials.gov identifier: NCT02063594).

The addition of nanoparticulate contrast agents containing inorganic contrast agents provide a unique opportunity for the visualization of a therapeutic agent, referred to as a theranostic. State-of-the-art clinical CT scanners already have the capability for acquiring the images with dual-energy by using either multiple layers of detectors or by employing rapid kilovolt switching from a single x-ray tube or different energy sources from dual source scanners (83). Visualization of materials is facilitated by the availability of modern clinical software equipped with capability for digital subtraction, effective anatomic number imaging and virtual monoenergetic reconstruction. Coupled with the use of novel inorganic agents these technologies can facilitate myocardial tissue characterization by allowing for material decomposition analysis. However, the human use inorganic contrast agents need to face some clinical challenges. Gold containing nanoparticles have been tested in clinical trials for cancer drug delivery demonstrating accumulation of gold nanoparticles in the tumor tissue, but also considerable liver uptake after administration with only about 50% elimination at 120 day after treatment (84). Importantly *in vivo* imaging of nanoparticles was not attempted in these trials, and current concentrations of the gold may be insufficient for *in vivo* imaging. Therefore, while molecular imaging with CT is a promising new avenue, this theranostic approach is not yet ready for widespread clinical application.

## Role of Multimodality Imaging in Evaluation of Anthracycline-Induced Cardiotoxicity

Anthracycline toxicity can have an insidious presentation, and therefore early recognition of the underlying disease process is very important to initiate preventive measures or to modify the anti-neoplastic therapeutic approach. In addition to the emerging role of CT, other imaging modalities can provide invaluable information about cardiac function that can help with management of AIC. Left ventricular ejection fraction (LVEF) assessment remains the key diagnostic parameter for the monitoring of anthracycline related cardiac dysfunction (85). Transthoracic echocardiography is the first line method for LVEF and strain evaluation (86), however cardiac MRI is emerging as the new gold standard of 3-dimensional quantification of global function and strain and has a growing role in the field (87). Equilibrium radionucleotide angiocardiography remains an accurate technique for LVEF assessment, although being used less frequently clinically for surveillance of cardiotoxicity due to concern of serial radiation exposure. In addition, the assessment of LVEF by CT and 3D echocardiography are newer alternatives for accurate assessment global function as well as regional function. Growing literature supports the use of echocardiographic LV strain to identify sub-clinical left ventricular dysfunction in patients undergoing anthracycline-based chemotherapy (27). CT and MRI provide alternative methods for the accurate and reproducible assessment of regional myocardial deformation strain. Therefore, a multimodality imaging approach is recommended and often applied.

## Conclusion

Cardiac CT is a rapidly evolving technology for the evaluation of cardiac structures with or without contrast administration, including the evaluation of the underlying coronary anatomy and/or complicating atherosclerotic or thrombotic disease. With emerging new techniques, CT is rapidly evolving to provide information beyond the evaluation of epicardial coronary arteries including myocardial deformation assessment, extracellular volume quantification, information about coronary vasoreactivity and even potential applications for molecular imaging. These new methodologies hold promise in the future for the early evaluation and management of AIC.

## Author Contributions

AF drafted the manuscript. LB and AS reviewed and edited the manuscript. All authors contributed to the article and approved the submitted version.

## Conflict of Interest

The authors declare that the research was conducted in the absence of any commercial or financial relationships that could be construed as a potential conflict of interest.

## Publisher’s Note

All claims expressed in this article are solely those of the authors and do not necessarily represent those of their affiliated organizations, or those of the publisher, the editors and the reviewers. Any product that may be evaluated in this article, or claim that may be made by its manufacturer, is not guaranteed or endorsed by the publisher.
